# Functional Characterization of Multiple *Ehrlichia chaffeensis* Sodium (Cation)/Proton Antiporter Genes Involved in the Bacterial pH Homeostasis

**DOI:** 10.3390/ijms22168420

**Published:** 2021-08-05

**Authors:** Lanjing Wei, Huitao Liu, Kimia Alizadeh, Maria D. Juarez-Rodriguez, Roman R. Ganta

**Affiliations:** Center of Excellence for Vector-Borne Diseases, Department of Diagnostic Medicine/Pathobiology, College of Veterinary Medicine, Kansas State University, New York, NY 66506, USA; lanjingwei@ku.edu (L.W.); hliu@vet.k-state.edu (H.L.); kimia@vet.k-state.edu (K.A.); mdjuarezro@icloud.com (M.D.J.-R.)

**Keywords:** functional genomics, rickettsial diseases, tick-borne diseases, antiporters, pH homeostasis

## Abstract

*Ehrlichia chaffeensis* causes human monocytic ehrlichiosis. Little is known about how this and other related tick-borne rickettsia pathogens maintain pH homeostasis in acidified phagosomes and the extracellular milieu. The membrane-bound sodium (cation)/proton antiporters are found in a wide range of organisms aiding pH homeostasis. We recently reported a mutation in an antiporter gene of *E. chaffeensis* (ECH_0379) which causes bacterial in vivo attenuation. The *E. chaffeensis* genome contains 10 protein coding sequences encoding for predicted antiporters. We report here that nine of these genes are transcribed during the bacterial growth in macrophages and tick cells. All *E. chaffeensis* antiporter genes functionally complemented antiporter deficient *Escherichia coli*. Antiporter activity for all predicted *E. chaffeensis* genes was observed at pH 5.5, while gene products of ECH_0179 and ECH_0379 were also active at pH 8.0, and ECH_0179 protein was complemented at pH 7.0. The antiporter activity was independently verified for the ECH_0379 protein by proteoliposome diffusion analysis. This is the first description of antiporters in *E. chaffeensis* and demonstrates that the pathogen contains multiple antiporters with varying biological functions, which are likely important for the pH homeostasis of the pathogen’s replicating and infectious forms.

## 1. Introduction

*Ehrlichia chaffeensis* is an obligate intracellular Gram-negative rickettsial pathogen and is the causal agent of monocytic ehrlichiosis in people and dogs [1,2,3]. The Lone-star tick, *Amblyomma americanum,* is identified as the biological vector for transmitting *E. chaffeensis* [4], while white-tailed deer is the major reservoir host [5]. *E. chaffeensis* has two developmental forms in infected vertebrate and tick host cells [6,7]: the smaller dense-cored cells (DCs) and the larger and pleomorphic reticulate cells (RCs). The DC form is released from the infected host cells and infects naïve host cells by phagocytosis. The DC form then transforms to the RC form, which replicates within the acidified phagosomes. RCs transform to DCs and are released from host cells by complete cell lysis or by exocytosis [6,7]. It is unclear how *E. chaffeensis* and other related rickettsia maintain pH homeostasis both within the acidified phagosomes and in the extracellular milieu where the pH is slightly basic [8]. 

Sodium/proton (Na^+^/H^+^) antiporters are integral membrane proteins required for maintaining the intracellular pH of cells in all living organisms, including in both Gram-positive and Gram-negative bacteria [9,10]. We previously described a function disruption mutation within the ECH_0379 gene of *E. chaffeensis* encoding for a Na^+^/H^+^ antiporter [11]. The mutation caused attenuated growth of the organism within its vertebrate hosts (deer and dogs), although the pathogen is not completely cleared from the hosts [11]. Further, we demonstrated the antiporter activity of the ECH_0379 gene product by utilizing a functional complementation assay in an *E. coli* strain with mutations in its antiporter genes [12]. In the current study, we extended our investigations to test the hypothesis that the *E. chaffeensis* genome contains multiple antiporters to facilitate the pathogen’s pH homeostasis during its intracellular growth in acidified phagosomes and the extracellular milieu. 

The biochemical characterization of *E. chaffeensis* proteins is challenging due to its obligate lifecycle and due to the lack of natural plasmids in the organism. Despite the recent substantial progress made so far in developing genetic approaches by our research team [11,12], biochemical characterization remains very limited for this and other related pathogens. In this study, we described the characterization of 10 predicted *E. chaffeensis* antiporter proteins by utilizing an *E. coli* mutant strain (EP432) with deficiency in its antiporter activity [12]. We used this *E. coli* strain in complementation experiments and defined the functions of all predicted *E. chaffeensis* antiporters. We further described differences in the functional *E. chaffeensis* antiporters, which may aid the pathogen’s pH homeostasis in acidic, neutral, and basic environments likely encountered during its growth in a vertebrate host.

## 2. Results

### 2.1. The E. chaffeensis Genome Has 10 Protein Coding Sequences Distributed across 6 Loci 

**Figure 1:**
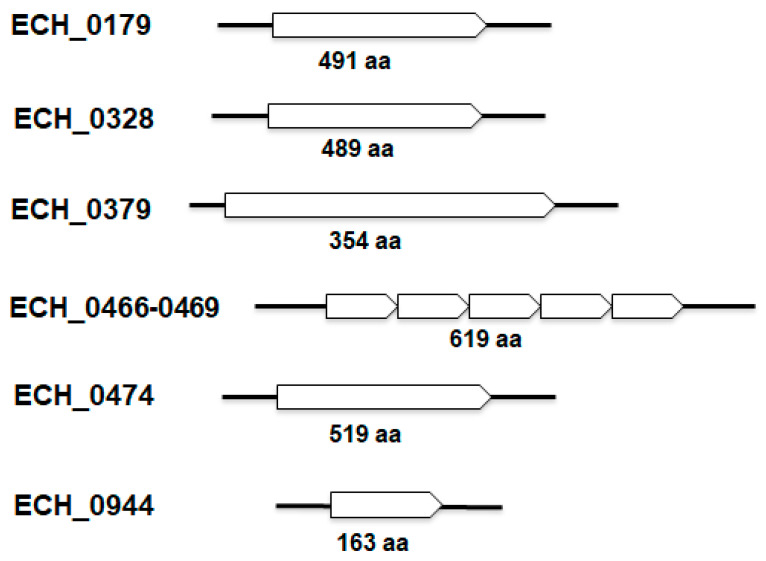
Antiporter genes identified from the *E. chaffeensis* genome. Gene names and GenBank accession numbers for the genome were included in Table 1.

Disruption mutation within the *E. chaffeensis* antiporter gene (ECH_0379) coding sequence caused reduced growth of the organism within its vertebrate hosts [11]. We hypothesized that the mutation caused the bacterial attenuation due to its inefficiency in regulating the pH homeostasis, while its survival at a low level in a host is the result of the functional redundancy of additional antiporters. Our analysis revealed the presence of several additional antiporter genes within the *E. chaffeensis* genome; we found 10 open reading frames (ORFs) coding for polypeptides identified as sodium (cation)/proton antiporter proteins or protein subunits (Table 1 and Figure 1). They are distributed across six loci. Five of the genes are found clustered in one locus. Based on the predicted annotation as antiporter protein subunits, we reasoned that the five-gene locus encoding subunits for one antiporter protein were made from a polycistronic message (Table 1). Thus, we considered the 10 ORFs as encoding six functional antiporter proteins. Homologs for all 10 genes are also present in other related Anaplasmataceae organisms of the genera, *Anaplasma, Ehrlichia* and *Wolbachia*. Secondary structure predictions analysis revealed that all 10 predicted antiporter polypeptides contain transmembrane domains (Figure S1, see Supplementary Materials).

### 2.2. E. chaffeensis Antiporter Transcription Assessed In Vitro

**Figure 2:**
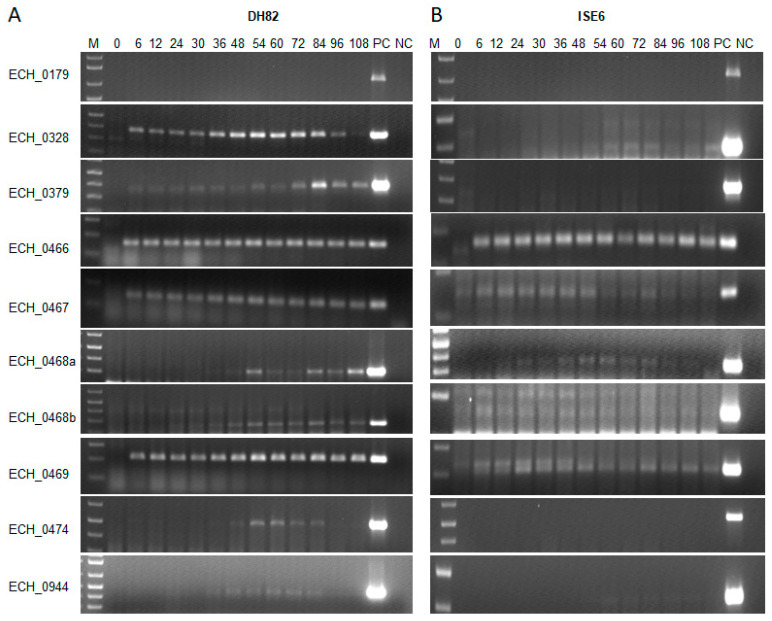
(**A**) Transcription assessed for the *E. chaffeensis* antiporter genes during the replication in macrophage (DH82) and (**B**) tick cell (ISE6) cultures. RNA concentration was measured by qRT-PCR targeted to the 16S rRNA and input RNA for each sample was normalized based on the 16S rRNA concentrations. The transcript levels for the targeted genes were then determined by semi-quantitative PCR with varying numbers of amplification cycles per target, which ranged from 25 to 40 cycles. The transcript levels were measured for different post-infection times: 0, 6, 12, 24, 30, 36, 48, 54, 60, 72, 84, 96, and 108 h. Positive controls (PC) for the assays included *E. chaffeensis* genomic DNA as the template. Negative controls (NC) had nuclease-free water as the template. The lowest number of temperature cycles where we noted the expression for each gene are presented: ECH_0179 (45 cycles), ECH_0328 (35 cycles for DH82 and 45 cycles for ISE6), ECH_0379 (35 cycles for DH82 and 45 cycles for ISE6), CH_0466 (35 cycles for DH82 and 40 cycles for ISE6), ECH_0467 (35 cycles for DH82 and 45 cycles for ISE6), ECH_0468a (5′) (45 cycles for DH82 and 35 cycles for ISE6), ECH_0468b (3′) (35 cycles for DH82 and 45 cycles for ISE6), ECH_0469 (30 cycles for DH82 and 45 cycles for ISE6), ECH_0474 (35 cycles for DH82 and 45 cycles for ISE6), and ECH_0944 (45 cycles).

To assess if the antiporter ORFs were actively transcribed, *E. chaffeensis* RNAs recovered during the bacterial active in vitro replication within a macrophage cell line (DH82) and tick cell line (ISE6) was evaluated by semi-quantitative RT-PCR using primer pairs targeting all 10 ORF (Figure 2). Except for the ECH_0179 ORF, all other ORFs tested positive for transcripts in RNA recovered from macrophage cultures. The RNA expression for ECH_0466, ECH_0467, and ECH_0469 was very similar and remained constant during bacterial growth in the vertebrate macrophage cultures. The remaining genes had varying levels of expression, which increased with bacterial growth over time, and higher transcription was observed between 54 h and 84 h. As the replicating form (RC) peaks at around the mid-infection phase, we reasoned that the increased transcription for some of the antiporters represents the active demand for the proteins during bacterial replication within infected phagosomes. The gene expression patterns were similar for the *E. chaffeensis* cultured in tick cells, except that the expression levels for ECH_0328, ECH_0379, ECH_0474, and ECH _0944 were lower compared to the gene expression in macrophage cultures. As in the macrophage cultures, RNA for the ECH_0179 ORF was undetectable in tick cell culture.

### 2.3. Transcription of E. chaffeensis Antiporter Genes Assessed in the Antiporter Function Deficient E. coli Mutant (EP432)

**Figure 3:**
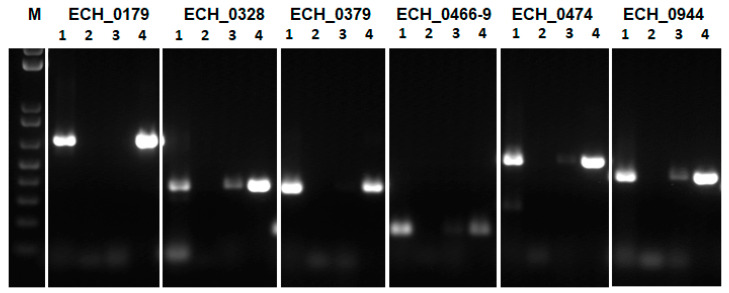
*E. chaffeensis* antiporter gene transcripts assessed by RT-PCR for the respective gene recombinant plasmids transformed into the *E. coli* strain EP432. The assays for each gene included four reactions: 1, RNA sample with reverse transcriptase and Taq polymerase; 2, nuclease-free water with reverse transcriptase and Taq polymerase (to serve as the negative control); 3, RNA template with Taq polymerase and without reverse transcriptase (to serve as the negative control and to eliminate false positives resulting from the residual DNA contamination; 4, *E. chaffeensis* genomic DNA with Taq polymerase (to serve as the positive control). (Note; weaker amplicons found in lane 3 for ECH_0328, ECH_0466-9, ECH_0474 and ECH_0944 represent the incomplete elimination of DNA contamination).

Since molecular tools are still limited for use in the Anaplasmataceae family pathogens, including for *E. chaffeensis*, it is challenging to study gene/protein function in this pathogenic bacterium in vivo. To overcome this limitation, we used *E. coli* as a surrogate system to study the functions of genes and proteins of *E. chaffeensis* [12,13]. Recently, we reported an *E. coli* function complementation system to define *E. chaffeensis* antiporter protein gene transcription and protein function [12] (Figure S2). We described that the functional complementation studies in the *E. coli* EP432 strain are ideally suited for defining the function of *E. chaffeensis* antiporter genes as it has mutations in two of its native antiporter genes [12,14]. The *E. coli* EP432 strain growth is significantly inhibited in the presence of sodium chloride [14] and the growth can be restored with its native gene (*nhaA*) expressed from a recombinant plasmid or with another bacterial gene homolog that it can functionally complement [12,15,16,17]. To define the functions of *E. chaffeensis* antiporter genes, we transformed the EP432 strain with recombinant plasmids containing *E. chaffeensis* antiporter genes cloned along with their predicted promoter segments; this was conducted one gene at a time. The *E. coli nhaA* gene was similarly cloned to serve as a positive control. All transformed EP432 containing *E. chaffeensis* antiporter gene recombinant plasmids were then assessed for transcripts by RT-PCR analysis (Figure 3). Predicted amplicons were detected in the transformed EP432 organisms containing the *E. chaffeensis* antiporter genes.

### 2.4. E. chaffeensis Antiporter Proteins Functionally Complement Antiporter Deficiency in E. coli

**Figure 4:**
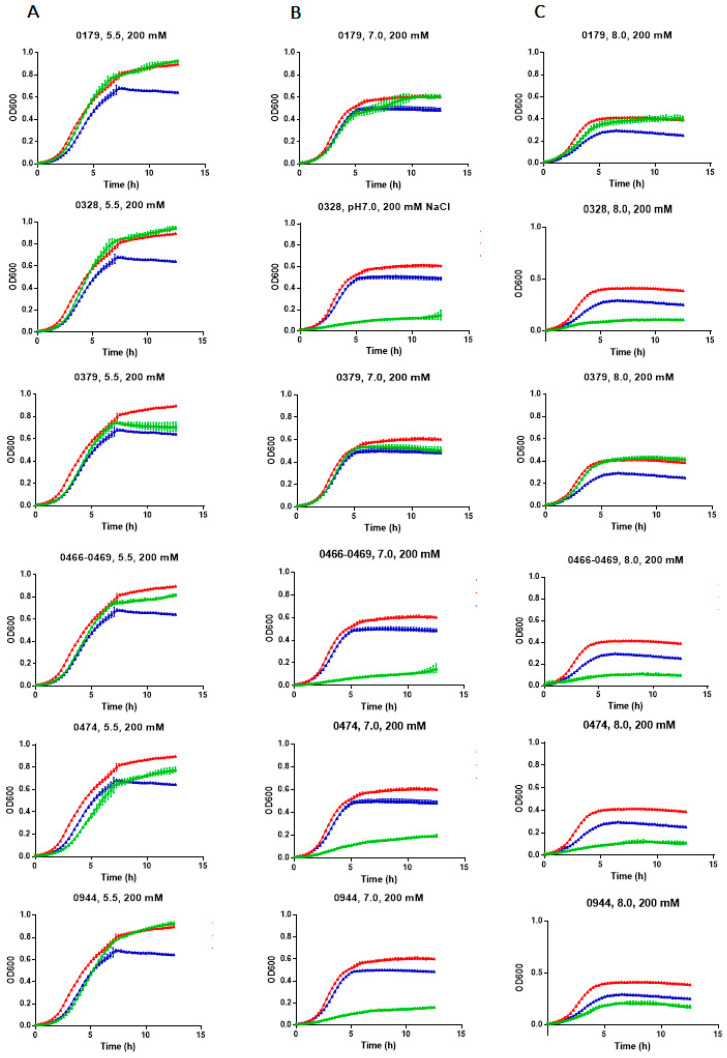
*E. chaffeensis* antiporters restored the growth of a salt-sensitive sodium/proton antiporter deficient strain of *E. coli*. The growth curves were measured by monitoring the OD at 600 nm over a period from 15 min to 13 h. The salt-stress was induced by the inclusion of 200 mM NaCl in LB media at varying pH conditions—5.5, 7.0, or 8.0—for all *E. chaffeensis* antiporter gene-containing EP432 strains (green lines). The *E. coli* transformed with the non-recombinant plasmid (blue line) or the plasmid-containing *E. coli* antiporter gene (*nhaA*) (red line) served as negative or positive controls, respectively. *E. coli* growth curves assessed at pH 5.5 (**A**), 7.0 (**B**) and 8.0 (**C**) are presented.

We then evaluated if *E. chaffeensis* antiporter proteins are synthesized in EP432 and reversed the growth inhibition of EP432 in the presence of 200 mM NaCl at varying pH conditions of the culture media. The complementation at pH 5.5 was observed for all 6 genes (Figure 4 A; green lines). The ECH_0179, ECH_0328, and ECH_0944 proteins complemented *E. coli* growth very similarly to that observed with its native *nhaA* gene (positive control) (Figure 4; red lines in each image). Complementation with the ECH_0466-0469 gene cluster was slightly less compared to ECH_0179, ECH_0328, and ECH_0944 proteins, and weaker for the ECH_0379 and ECH_0474 proteins. The *E. coli* growth restoration was also observed for ECH_0179 protein at both neutral and basic pH conditions, whereas the ECH_0379 gene complemented at pH 8.0 with no change at neutral pH (Figure 4 B and C). In contrast, genes ECH_0328, ECH_0466-0469, ECH_0474, and ECH_0944 made the *E. coli* more sensitive to NaCl growth at pH 7.0 and pH 8.0.

### 2.5. Native ECH_0379 Formed a Dimer and Expressed in Both Replicating (RC) and Infectious (DC) Forms and Similarly It Forms Dimers When Expressed as a Recombinant Protein

As ECH_0379 protein had the antiporter activity in a broad pH range, we hypothesized that it is expressed in both RC and DC forms of *E. chaffeensis*. 

**Figure 5:**
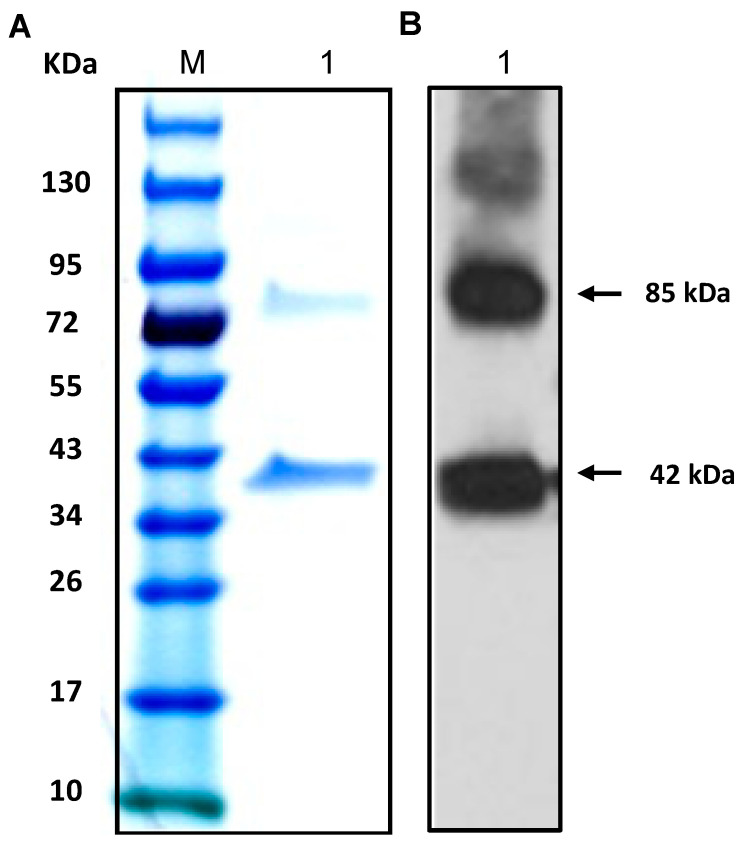
ECH_0379 recombinant protein purified to homogeneity using the pET expression system in *E. coli*. (**A**) The purified recombinant protein was resolved by 12% SDS-PAGE and stained with Coomassie blue G250 stain. (**B**) Western-blot analysis performed for the SDS-PAGE resolved proteins using the N-terminal expressed His-tag-specific polyclonal sera. (M, protein molecular weight markers; 1, resolved recombinant protein).

**Figure 6:**
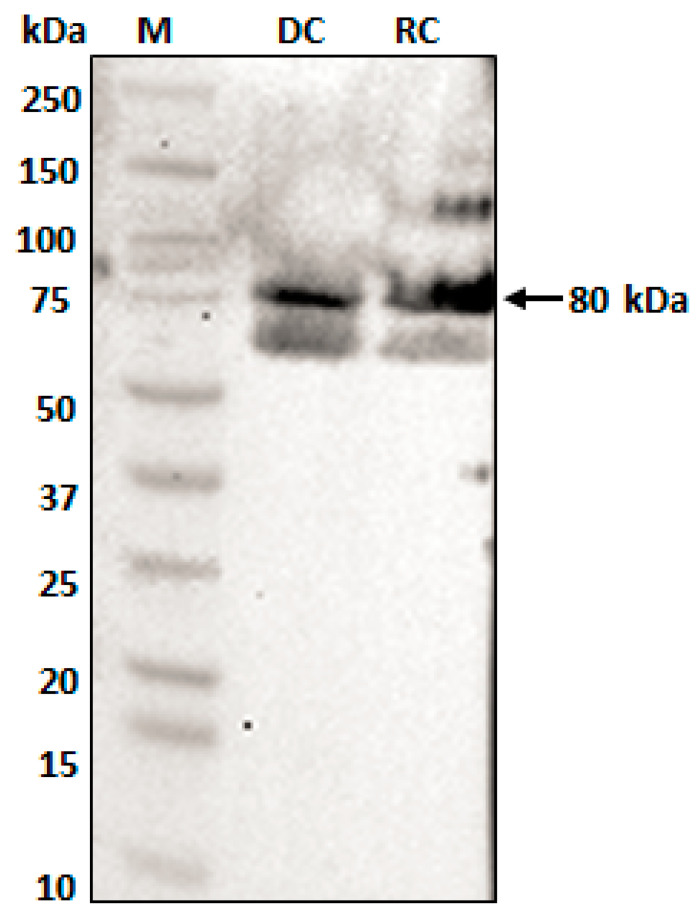
ECH_0379 antiporter expression assessed in RC and DC forms of *E. chaffeensis* cultured in vitro. Density gradient-purified fractions of RC and DC forms were used to recover *E. chaffeensis* proteins and were subjected to Western blot analysis using polyclonal sera raised against ECH_0379 recombinant protein. (M, protein molecular weight markers; DC and RC refer to purified *E. chaffeensis* proteins recovered from DC and RC fractions, respectively).

To test this hypothesis, we first cloned and expressed the ECH_0379 recombinant protein in *E. coli* using a pET expression system and purified to homogeneity (Figure 5). The recombinant protein migrated as two different molecular masses: about 42 kDa and 85 kDa with His-tag. The 42 kDa size was the expected size for the His-tagged recombinant protein, while the second larger protein is likely the self-association of ECH_0379 protein forming dimers. The presence of both bands as part of the recombinant protein was confirmed by Western blot analysis using His-tag antibodies. Further, mass-spectrometry analysis by MALDI-TOF (Matrix-Assisted Laser Desorption/Ionization Time-of-Flight) confirmed that both protein bands contained the ECH_0379 protein sequence (not shown). The dimer formation of ECH_0379 antiporter is similar to other known antiporter proteins [16,18]. Antisera was then raised against the recombinant ECH_0379 antiporter and subsequently used to assess protein expression in the RC and DC forms of *E. chaffeensis* (Figure 6). One protein band of 80 kDa size that is equivalent to a dimer was recognized by the ECH_0379 antisera in the proteins recovered from both the RC and DC forms of *E. chaffeensis.*

### 2.6. Proteoliposome Prepared with the Recombinant ECH_0379 Protein Assessed in Diffusion Analysis

**Figure 7:**
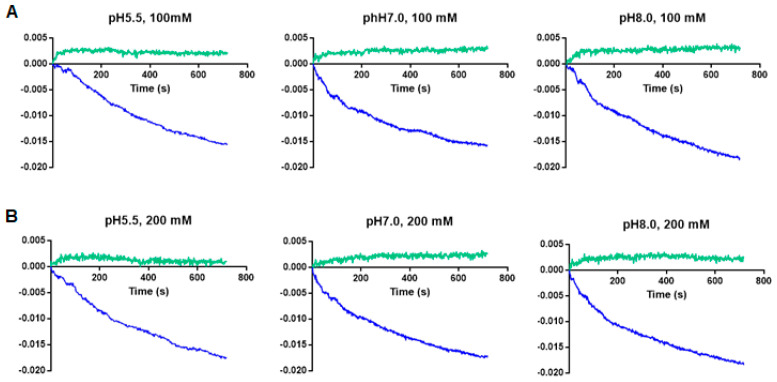
The NaCl diffusion assays performed using the proteoliposomes constituted with the ECH_0379 recombinant protein in lipid vesicles. The diffusion rates were measured by monitoring the decrease in OD 400 nm absorbance in the presence of NaCl at 100 mM (**A**) or 200 mM (**B**) concentrations and at three different pH conditions. Blue and green lines represent liposomes prepared with and without the purified ECH_0379 recombinant protein, respectively. The data represent the average values from three independent experiments.

To independently verify the antiporter activity of *E. chaffeensis* ECH_0379 antiporter protein, we performed in vitro proteoliposome diffusion assays using the recombinant protein. The recombinant protein was used to constitute proteoliposomes and tested in proteoliposome assays (Figure 7). A steady decline of the absorbance at 400 nm was observed in the presence of NaCl when tested at two different concentrations—100 mM or 200 mM—and at three different pH conditions (pH 5.5, 7.0, and 8.0) (Figure 7; blue lines). In control experiments where the recombinant protein was not included in the proteoliposome preparations, there was no change in the absorbance at 400 nm in the solutions containing either 100 mM or 200 mM NaCl independent of the pH variations (negative controls) (Figure 7; green lines).

## 3. Discussion

Sodium (cation)/H+ antiporters facilitate the exchange of protons for sodium or other cations [19,20,21]. Antiporters are critical in regulating intracellular pH, sodium levels, and cell volume [22,23]. Antiporters are found in diverse organisms [24,25,26,27,28,29]. *E. chaffeensis* is an important tick-transmitted obligate pathogenic bacterium causing diseases in humans and dogs [30,31]. This intraphagosomal pathogen replicates within acidified vacuoles, while the infectious form is exposed to a physiological pH of ~7.0. The pathogen’s ability to survive in a phagosome environment is remarkable, as it requires efficient antiporters for pH homeostasis to maintain its physiological pH. Currently, no studies have described how the pathogen and other related intraphagosomal rickettsial pathogens maintain their cytoplasmic pH homeostasis.

*E. chaffeensis* transitions between the infectious form (DC) outside a host cell where the pH of the environment is expected to be ~7, while its replication takes place within acidified phagosomes of monocytes/macrophages. Thus, both the intracellular replicative form (RC) and the extracellular DC form of *E. chaffeensis* require adaptation to rapidly shifting ionic strengths and pH. This may be best accomplished by multiple antiporters with diverse biological functions in support of the pathogen’s lifecycle within a host. We recently reported that a functional disruption mutation in an *E. chaffeensis* antiporter gene (ECH_0379) causes considerable decline of the pathogen growth within vertebrate hosts [11,32]. Despite the antiporter gene function deficiency, the mutant remained in the hosts, although the rickettsemia levels fell below detectable levels in circulation as compared to wild type *E. chaffeensis*-infected animals [11,32]. These data suggest that antiporter functional redundancy exists in the organism to support its pH homeostasis. Bacterial pathogens are known to contain different types of antiporters expressed on the membranes to support their growth in diverse environmental conditions [17,33,34,35]. The results presented in the current study represent the first detailed description of multiple antiporters. Our initial search in the *E. chaffeensis* genome allowed the identification of 10 gene open reading frames encoding for sodium (cation)/proton antiporter proteins or protein subunits. Homologs for all antiporter genes are also present in the genomes of other *E. chaffeensis* strains, as well as in all Anaplasmataceae family organisms of the genera *Ehrlichia, Anaplasma,* and *Wolbachia* for which the genome sequences are available in the GenBank. Further, analysis of the transmembrane topology revealed that all encoded antiporter proteins from the genes possess transmembrane domains. Except for one, all genes were also transcribed in *E. chaffeensis* during its replication in macrophage and tick cell cultures. We then assessed the antiporter genes in *E. coli* mutant with functional deficiency for antiporters; we presented data demonstrating that all predicted antiporters are functionally active. Specifically, our study demonstrated that all *E. chaffeensis* genes can complement the *E. coli* antiporter function at pH 5.5, while some genes can also provide complementation at physiological pH conditions. The data establish that all putative *E. chaffeensis* antiporter genes code for functional sodium/proton antiporters.

Transcriptional-level expression analysis of RNA transcripts of the putative antiporter protein genes of *E. chaffeensis* were detected when cultured in the macrophage cell line and tick cells, suggesting that they are functional genes and that the translated proteins of the antiporters may have a biological function during its lifecycle within its vertebrate and tick hosts. In the *E. coli* surrogate system, recombinant proteins made from all predicted antiporters provided functional complementation in relieving the inhibition of its growth in the presence of NaCl at pH 5.5. In contrast, the protein expression at neutral pH was unfavorable for all gene products except for ECH_0179 and ECH_0379, thus making the EP432 strain of *E. coli* more sensitive to NaCl. ECH_0379 provided the most complementation at pH 8.0, with minor impact at acidic pH and no major impact at neutral pH. These data suggest that the ECH_0379 protein may be critical for the bacterium during its growth within the phagosomes and after its release as DC. Indeed, our data demonstrate that this protein is expressed in both RC and DC forms of *E. chaffeensis.* Antiporters synthesized from the ECH_0179 and ECH_0379 genes, therefore, are likely to support the bacterial homeostasis for RC and DC forms. ECH_0179 RNA expression was undetectable when the organism was grown in either macrophage or tick cell cultures, while the ECH_0379 expression was observed at all time points assessed. Consistent with this observation, the ECH_0379 protein was detected in both the RC and DC forms of *E. chaffeensis*. The lack of detectable RNA transcripts for the gene ECH_0179 may indicate that it may have low expression under *in vitro* culture conditions, while it is likely expressed in vivo. Together, ECH_0379 and ECH_0179 gene products may help the bacterium to maintain the pH homeostasis for its growth within a phagosome and during its presence outside a host cell. More studies are necessary to define how such functional diversity contributes to bacterial in vivo growth. Our studies are consistent with prior reports on antiporters of other Gram-negative bacteria, where researchers reported that the sodium/proton antiporters have characteristic individual pH-dependent activity profiles, critical for their homeostatic functions [36,37]. 

SDS-PAGE and the Western blot analysis of the purified recombinant protein ECH_0379 revealed two resolved proteins with different molecular weights. The fastest migrating protein was about 42 kDa and the higher molecular mass protein was about 85 kDa, which is twice the size of the predicted molecular weight, suggesting that it represents a dimerized protein. When assessed for the native protein using the ECH_0379 polyclonal sera, we observed only one larger dimerized protein band, which migrated at ~80 kDa. A similar dimer formation has been reported previously for antiporters of *E. coli* [18] and *Salmonella* [16]. The purified recombinant ECH_0379 antiporter protein reconstituted as lipid vesicles (proteoliposomes) demonstrated antiporter activity under the three different pH conditions and at two different NaCl concentrations. These data validate that the ECH_0379 antiporter indeed has the antiporter function and may be critical for the bacterial pH homeostasis in a host. 

**Figure 8:**
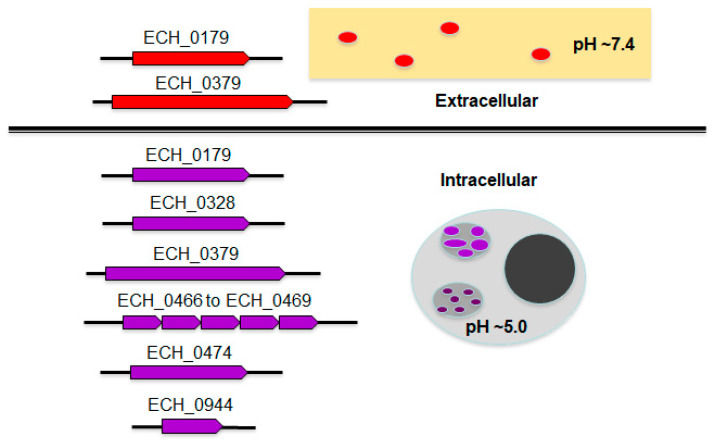
Model cartoon outlining *E. chaffeensis* antiporters for their possible roles in pH homeostasis in the *Ehrlichia*-containing acidified phagosomes and during the release of the DC form in the extracellular milieu.

The current study is the first and most detailed description of antiporters in the rickettsial organism: *E. chaffeensis*. The pathogen contains multiple genes encoding for functional antiporters which fall under two sub-groups based on their biological properties (Figure 8). While all encoded antiporters support the growth of the organism within the acidified phagosomes, a subset of proteins may also aid the organism’s pH homeostasis during its exposure to the cell-free environment where the infectious DC form may migrate to enter naïve macrophages in vivo via systemic spread. Thus, the expression of all *E. chaffeensis* antiporters is likely critical in supporting the bacterial ion balance to regulate its internal pH. The pathogen’s DC form is released out of a host cell where pH is likely to be close to between 7.35 and 7.45 [8], and ECH_0179 and ECH_0379 proteins may facilitate pH homeostasis of the organism. 

This research marks the beginning the advancement of our understanding of how *E. chaffeensis* and other related rickettsia regulate their intracellular pH under diverse host environments, where the pathogens are exposed to acidic and physiological pH conditions. Notably, we discovered, following BLAST search analysis, that the multiple antiporter gene homologs are also well conserved in other related *Anaplasma* and *Ehrlichia* species pathogens. Considering that therapeutics are limited to only one class of antibiotics (tetracycline derivatives) for treating rickettsial diseases [38,39], proteins that mediate the pH homeostasis can serve as ideal targets for developing alternate drug choices. Indeed, antiporters are well-established as drug targets for bacterial infections [40,41]. Thus, defining the contributions of antiporters in *Ehrlichia* species and other related obligate rickettsial pathogens will also aid in advancing the research on their interference with bacterial growth *in vivo*. Studies can now be extended to further characterize *E. chaffeensis* antiporters for their role in the pathogen’s life cycle and result in the prospects for developing novel treatment options for the HME and other tick-borne rickettsial diseases in humans and animals.

## 4. Materials and Methods

### 4.1. Bioinformatic Analysis 

The identity of 9 new antiporter gene open reading frames was established based on the existing annotation data for the *E. chaffeensis* genomes entries: GenBank accession numbers NC_00779 and CP000236. We previously reported that ECH_0379 gene coding sequence has a significant homology with bacterial antiporter protein subunits (Cheng et al. 2013). The BLAST search analysis identified homologs for all 10 *E. chaffeensis* antiporters in other related Anaplasmataceae family bacteria of the genera *Anaplasma, Ehrlichia,* and *Wolbachia*, for which whole genome sequences are available in the GenBank database. The secondary structure predictions of the *E. chaffeensis* putative antiporter proteins were performed using the online sequence analysis tools on 2–6 April 2018: YASPIN (http://www.ibi.vu.nl/programs/yaspinwww/) and Smart (http://smart.embl-heidelberg.de/). TMpred was used for transmembrane domain predictions (https://www.ch.embnet.org/software/TMPRED_form.html). Searches for homologous protein sequences were performed using the SIB BLAST+ Network Service (https://web.expasy.org/blast/). Conserved domains within the putative antiporter proteins were performed at https://www.ncbi.nlm.nih.gov/Structure/cdd/wrpsb.cgi. Multiple sequence alignments for all the identified *E. chaffeensis* predicted antiporter proteins and homologous antiporter protein sequences were conducted using Clustal Omega (https://www.ebi.ac.uk/Tools/msa/clustalo/).

### 4.2. Cultivation of E. chaffeensis

*E. chaffeensis* Arkansas isolate (ATCC # CRL-10389) was cultured in the canine macrophages cell line (DH82) or in the *Ixodes scapularis* embryonic cell line (ISE6) as previously described [12,42]. For the RNA expression analysis experiments, infected cultures were recovered at various time points post-infection from 0 to 108 h: 0, 6, 12, 24, 30, 36, 48, 54, 60, 72, 84, 96 and 108 h. The harvested cells from each flask were concentrated by centrifugation at 12,000 g for 10 min. Supernatants were discarded, and the final pellets were re-suspended in 1 mL of TRI-Reagent (Sigma-Aldrich, St. Louis, MO) and stored at −80 °C until RNA isolation was completed.

### 4.3. RNA Isolation 

Total RNA was extracted using the TRI-Reagent method (MilliporeSigma, St. Louis, MO, USA), as per the manufacturer’s recommended protocol, with the following minor modifications. The cell pellets dissolved in TRI-Reagent were thawed at room temperature and 0.2 mL of chloroform was added per 1 mL in each of the TRI-Reagent solutions. Then, the mixture was vortexed vigorously for 15 s, left to stand for 15 min at room temperature, and then centrifuged for 15 min at 12,000 g at 4 °C. The upper aqueous phase was transferred to a clean RNase-free tube, to which 0.5 mL of pre-cooled 2-propanol was added per ml of TRI reagent, then the solution was incubated for 10 min at 4 °C. RNA was recovered by centrifugation for 10 min at 12,000 g at 4 °C. The supernatant was discarded, and the pellets were rinsed with 1 mL of 75% pre-cooled ethanol per each ml of TRI reagent solution containing RNA. The solutions were mixed by pipetting up and down and then centrifuged at 12,000 g for 5 min at 4 °C. Supernatants were discarded; the RNA pellets were air-dried and resuspended in 50 μL of nuclease-free water. Residual genomic DNAs from each RNA were eliminated by adding RQ1 RNase-Free DNase (Promega, Madison, WI, USA) as per the manufacturer’s protocol and stored at −80 °C until use.

### 4.4. Gene Expression Levels Determined by Semi-Quantitative PCR 

RNA samples from the different time points post-infection from in vitro cultures were normalized following defining the 16S rRNA levels by TaqMan probe-based RT-PCR assay, as described in a previous study [43], using the gene-specific rimers and probe, and using the commercial reagent kit; SuperScript^®^ III Platinum^®^ One-Step Quantitative RT-PCR System (Invitrogen, Carlsbad, CA, USA). Additional details pertaining to the assay conditions, including the primers and probe used for the assay and standard curve assessment, are described in [43]. The amplification protocol included a reverse transcription step for 30 min at 50 °C, followed by 5 min of denaturation at 95 °C, and 40 cycles of amplification performed for 15 s at 95 °C, 40 s at 50 °C, and 45 s at 60 °C with the optics setting turned on in the StepOnePlus real-time PCR system (Thermo Fisher, Waltham, MA, USA). Based on the 16S qRT-PCR results, all the RNA concentrations were adjusted to have an equal copy number of 16 S rRNA per unit volume of RNA. The nucleotide sequences of the antiporter genes are available at the GenBank {available online: https://www.ncbi.nlm.nih.gov/ (28 July 2021)}. Primers for the one-step RT-qPCR were designed from within the coding sequences and synthesized by Integrated DNA Technologies (Coralville, IA, USA). The nucleotide sequences of the primers are listed in Table 2. Primers targeted to *E. chaffeensis* antiporter genes were then used to perform one-step reverse transcription PCR (RT-PCR) using 2 μL of each of normalized RNA templates with the SuperScript^®^ III Platinum^®^ One-Step Quantitative RT-PCR System commercial kit (Invitrogen, Carlsbad, CA, USA). The amplification conditions for the assays were as follows: reverse transcription for 1 h at 50 °C, then amplification cycles set at 30 s at 94 °C and 30 s at 51 °C for genes ECH_0328, ECH_0466, ECH_0467, ECH_0468a, EHC_0468b, EHC_0469, and ECH_0944) or 54 °C for genes ECH_0179, ECH_0474, and ECH_0379, then 30 s at 72 °C followed by a final extension step for 5 min at 72 °C, and then storage at 4 °C until analysis. The amplification cycles varied from 25 to 45 cycles to estimate the variations of gene transcription levels. The amplicons were resolved in 1.5% agarose gel containing ethidium bromide, and images of the DNA resolved in the gels were captured using a Kodak Gel Logic 200 imaging system.

### 4.5. Constructing the Recombinant Plasmids

Based on the location of the predicted antiporter genes of the *E. chaffeensis* genome, five ORFs spanning from ECH_0466 to ECH_0469 were considered as encoding for one antiporter protein transcript made from one promoter segment located upstream to the first gene (ECH_0466), thus reducing the total number of predicted genes to 6 (Figure 1). The coding sequence of each gene along with the respective promoter sequence was amplified by PCR using a high-fidelity DNA polymerase (Invitrogen, Carlsbad, CA) with the primer sequences listed in Table 3, and using *E. chaffeensis* genomic DNA as the template. PCR products were then cloned into the plasmid, pBluescript II SK (+) (Stratagene, San Diego, CA) by directional cloning into *Bam*HI and *Sal*I restriction enzyme sites generated as part of the amplicons. All the restriction enzymes used in the experiment were bought from NEB (New England Biolabs, Ipswich, MA). The coding sequence of the *E. coli* antiporter gene (*nhaA*; GenBank accession number NJ74_RS08715) with its promoter sequence was similarly cloned into pBluescript II SK(+) to serve as a positive control. The presence of specific inserts in each of the recombinant plasmids was confirmed by DNA sequence analysis. The recombinant plasmids were named pBSK-0179, pBSK-0328, pBSK-0379, pBSK-0466-0469, pBSK-0474, and pBSK-0944 for *E. chaffeensis* antiporter genes, and the *E. coli nhaA* gene recombinant plasmid was named pBSK-NhaA.

The pET-28(+) vector (Novagen, Darmstadt, Germany) was used to clone the ECH_0379 gene coding sequence for its protein expression and purification by including the N-terminal His-tag as part of the recombinant protein. The entire protein-coding sequence of ECH_0379 was amplified by PCR using *E. chaffeensis* genomic DNA as the template and using the proofreading enzyme, pfu DNA polymerase (Promega, Madison, WI). ECH_0379 ORF-specific PCR primers were designed to include *Nhe*I and *Xho*I restriction sites on the forward and reverse primers, respectively. The PCR products were cloned into pET28 plasmid at *Nhe*I and *Xho*I to generate the recombinant expression plasmid by following standard molecular cloning procedures, with initial cloning performed utilizing the *E. coli* TOP10 strain to generate the recombinant plasmid (pET28-ECH_0379). Following verification of the insert orientation by restriction enzyme digestion analysis and also by DNA sequence analysis, the plasmid was retransformed into the *E. coli* BL21 (DE3) strain (Invitrogen, Carlsbad, CA) for use in recombinant protein synthesis.

### 4.6. Growth Complementation under NaCl Stress

Antiporter activities of the putative *E. chaffeensis* antiporters were assessed by performing functional complementation assays in the *E. coli* strain, EP432. The antiporter gene-containing recombinant plasmids and the *E. coli nhaA* gene-carrying plasmid were transformed into the EP432 strain; this was conducted one at a time. The *nhaA* gene-carrying plasmid transformed *E. coli* served as the positive control, while non-recombinant pBluescript II SK^+^ plasmid transformed *E. coli* served as the negative control. Before investigating the antiporter activities of the *E. chaffeensis* genes, we verified the RNA expression from each transformed *E. coli*; total RNA was recovered from 3 mL each of overnight bacterial cultures of recombinant *E. coli* grown in LB media (10 g/L tryptone, 5 g/L yeast extract, and 200 mM NaCl) and was used for the analysis. After the DNase treatment, RNAs were assessed by one-step RT-PCR following the same protocol described in the previous section but the amplification cycles were set to 40. The PCR products were analyzed in a 1.5% agarose gel and the DNA images were captured.

Upon transcription verification of all *E. chaffeensis* putative antiporter genes and the *E. coli* antiporter gene, and their absence in *E*. *coli* being transformed with the non-recombinant plasmid, all EP432 transformed cultures were used to test the antiporter activities. Overnight cultures of transformed EP432 *E. coli* strains were grown in LBK medium (10 g/L tryptone, 5 g/L yeast extract, and 6.5 g/L KCl). The cultures were then diluted to 0.02 optical density units at 600 nm (OD_600nm_) in LB (10 g/L tryptone, 5 g/L yeast extract) media containing 200 mM NaCl with the media pH adjusted to three different pHs: 5.5, 7.0, or 8.0. For pH 5.5 conditions, 100 mM 2-(N-morpholino) ethanesulfonic acid (MES) buffer at pH 5.5 was used at the final concentration in the LB media, while for the pHs 7.0 and 8.0, 100 mM Tris-HCl buffer was used at the desired pHs. The cultures in microwell plates were incubated in a Microbiology Reader Bioscreen C (Oy Growth Curves Ab Ltd., Helsinki, Finland) at 37 °C with continuous shaking. The growth in the wells of the culture plates was monitored once every 15 min by measuring OD_600nm_ for 13 h. The experiment was performed three independent times.

### 4.7. Overexpression and Purification of the Protein Encoded by ECH_0379

The ECH_0379 protein was overexpressed in *E. coli* BL21 (DE3) strain transformed with pET28-ECH_0379 plasmid. The induction in the exponential growth phase (OD_600nm_ = 0.5) was initiated by the addition of 1 mM isopropyl-β-D-thiogalactopyranoside (IPTG) and the cultures were maintained at 30 °C in a shaking incubator. Cells were harvested after 12 h of cultivation and stored at −80 °C. The histidine tagged ECH_0379 protein product was purified using Ni-NTA-agarose (Qiagen, Germany). The cell pellet was suspended in 10 mL of lysis buffer and incubated for 60 min on ice with gentle mixing. After sedimentation of cell debris, 2 mL Ni-NTA-agarose slurry was added to the lysate. The mixture was incubated for 1 h at 4 °C, and then loaded on a poly-prep chromatography column (Bio-Rad, Hercules, CA, USA). The desired protein was eluted using 1x elution buffer (50 mM sodium phosphate pH 8.0; 80 mM imidazole; 137 mM NaCl; 10% (*v*/*v*) glycerol), collected as 1 mL fractions, and analyzed in a 12% sodium dodecyl sulfate-polyacrylamide gel electrophoresis (SDS–PAGE). The protein concentration was determined using the Bio-Rad protein assay kit (Hercules, CA, USA) as per the manufacturer’s recommended protocol. The integrity of the recombinant protein, as made from ECH_0379 gene, was confirmed by mass-spectrometry by subjecting it to MALDI-TOF analysis using a fee-for-service facility (biotechnology/proteomics core laboratory at Kansas State University).

### 4.8. Western Blot Analysis to Detect ECH_0379 Protein Prepared in E. coli as Well as the Native Protein Expressed in RC and DC Purified Fractions of E. Chaffeensis

For the detection of recombinant ECH_0379 protein, the above-described SDS-PAGE resolved proteins were transferred onto a nylon membrane (Thermo Fisher Scientific, Waltham, MA, USA) by subjecting them to electro-blotting using an electrophoretic transfer unit (Bio-Rad, Hercules, CA, USA). Subsequently, the presence of recombinant protein was assessed using a His-tag antibody as per the manufacturer instructions (Thermo Fisher, Rockford, IL, USA). The RC and DC forms of *E. chaffeensis* were purified by subjecting them to renografin density gradient centrifugation, as described previously [44]. About 20 µg each of the total *E. chaffeensis* proteins from the RC and DC fractions were resolved on an SDS-PAGE and transferred to a nylon membrane, as indicated above, and used for blot analysis with the ECH_0379-specific polyclonal antibody. Polyclonal serum was raised in rabbits against ECH_0379 recombinant protein using a fee-for-service facility (Thermo Fisher, Waltham, MA, USA). A secondary anti-rabbit antibody conjugated with horseradish peroxidase (Sigma-Aldrich, St. Louis, MO, USA) was used for the signal detection.

### 4.9. Proteoliposome Diffusion Assays

To determine the antiporter activity of ECH_0379 antiporter protein, proteoliposomes were constructed to perform the NaCl solution absorption assays, as described previously, with some minor modifications [45,46,47,48]. Two hundred and forty microliters of egg phosphatidylcholine (10 μmol/mL) (Avanti Polar Lipids, Alabaster, AL, USA) and 20 μL dicetylphosphate (10 μmol/mL) (Sigma-Aldrich, St. Louis, MO, USA) dissolved in chloroform-methanol (2:1 [vol/vol]) were mixed and dried under a stream of nitrogen gas for about 5 min in round bottom 5-SV borosilicate 5 mL tubes (Thermo Fisher, Waltham, MA, USA). Then, the lipid film was resuspended with 200 μL of 20 mM Tris-HCl buffer (pH 7.0) containing 2 μg of purified recombinant protein. The mixture was vortexed and homogenized by Branson water-bath M1800 sonicator (Branson Ultrasonic, Danbury, CT, USA) at room temperature until it became translucent and then the lipid-protein mixture was dried. We carefully added 300 μL suspension buffer (10 mM Tris-HCl, pH 7.5, 15% (wt/vol) dextran 40 (Sigma-Aldrich, St. Louis, MO, USA)) to the mixture. The tube was gently rotated to wet the film with incubating contents for 60 min. The mixture was then resuspended by gently shaking the tube. Seventeen μL of proteoliposome suspension was pipetted into 600 μL each of the three different testing buffer solutions—100 mM MES buffer at pH 5.5, 100 mM Tris-HCl buffer pH 7.0, or 100 mM Tris-HCl buffer at pH 8.0—containing 100 mM NaCl or 200 mM NaCl in a 0.1 cm quartz cuvette. Immediately after gentle mixing, the contents were pipetted up and down 5 times without producing air bubbles. Recordings were then taken at OD_400nm_ every 0.03 min (1.8 s) continuously for 12 min using a spectrophotometer (Varian, Palo Alto, CA, USA). In addition, liposomes with no purified recombinant protein added were similarly tested following the same protocol as proteoliposome, and these served as the negative controls.

## 5. Conclusions

The current study is the first and most detailed description of antiporters in *E. chaffeensis*. While all encoded antiporters support the growth of the organism within the acidified phagosomes, a subset of proteins appear to also aid the organism’s pH homeostasis during its exposure to the extra cellular environment at physiological pH. This research marks the beginning of the advancement of understanding of how *E. chaffeensis* and other related rickettsia regulate their intracellular pH under diverse host environments. Further, considering that therapeutics are limited to only one class of antibiotics, proteins that mediate the pH homeostasis can serve as ideal targets for developing alternate drug choices. Thus, defining the contributions of antiporters in *Ehrlichia* will likely aid in advancing the research on their interference with bacterial growth in vivo.

## Figures and Tables

**Table 1:** Putative antiporters in *E. chaffeensis*.

Old_Locus_Tag *	New_locus_Tag ^$^	Length of AA ^#^	Function Annotation
ECH_0179	ECH_RS00755	491	cation/proton antiporter
ECH_0328	ECH_RS01335	489	cation/proton antiporter subunit D
ECH_0379	ECH_RS01545	353	hypothetical protein (sodium/proton antiporter; Cheng et al. 2013)
ECH_0466	ECH_RS01920	89	cation/proton antiporter subunit F
ECH_0467	ECH_RS01925	99	cation/proton antiporter subunit G
ECH_0468a (5′)	ECH_RS01930	181	DUF4040 domain-containing protein/sodium/proton antiporter subunit B (*E. ruminantium*)
ECH_0468b (3′)	ECH_RS01935	139	cation/proton antiporter subunit B
ECH_0469	ECH_RS01940	111	cation/proton antiporter subunit C
ECH_0474	ECH_RS01960	519	cation/proton antiporter subunit D
ECH_0944	ECH_RS03855	163	sodium/proton antiporter subunit E

* *E. chaffeensis* older genome accession number, CP000236. ^$^ *E. chaffeensis* newer genome accession number, NC_007799. ^#^ Predicted length in amino acids.

**Table 2:** Primers used for the semi qRT-PCR.

Gene	Primer Name	Sequence	Size
ECH_0179	RRG2107	Forward: 5′-GGCTATACAAGTTGGGTTGTTGT-3′	672 bp
	RRG2108	Reverse: 5′-CACACATACACCACAGATAGACCT-3′	
ECH_0328	RRG2068	Forward: 5′-GCATGCGATATCATTTGGAA-3′	370 bp
	RRG2069	Reverse: 5′-GAATTGGAAAAGCCGCATTA-3′	
ECH_0379	RRG1276	Forward: 5′-CTAAGGTTGTAGGGAATGCAACC-3′	374 bp
	RRG1277	Reverse: 5′-ACAAGGTAAGTACCTTGCTTGCTC-3′	
ECH_0466	RRG2054	Forward: 5′-TGCTGCAAATTTGTTTGGAA-3′	165 bp
	RRG2055	Reverse: 5′-TCTCCAAAAGAACCATGAAGA-3′	
ECH_0467	RRG2056	Forward: 5′-TGGACTTGCTATGCGATCTG-3′	151 bp
	RRG2057	Reverse: 5′-TCAGTCAGCATCATCTTACCTTT -3′	
ECH_0468a	RRG2062	Forward: 5′-TTTGATGGCATTGTTGCATT-3′	332 bp
	RRG2063	Reverse: 5′-CTCGAAAACTTGCTAAAATTGC-3′	
ECH_0468b	RRG2060	Forward: 5′-CAGGCTGGTGTTATTGTTGC-3′	259 bp
	RRG2061	Reverse: 5′-TACACACAGTCATGCCCACA-3′	
ECH_0469	RRG2058	Forward: 5′-GGTTATAGGTTTGTATGTTACTACTGC-3′	213 bp
	RRG2059	Reverse: 5′-ATTGCAACCCCAACAACAAT-3′	
ECH_0474	RRG2109	Forward: 5′-GGCATCTGGTGGGTTTTTAGG -3′	525 bp
	RRG2110	Reverse: 5′-GCAGAACATACTGCCTCTACTG-3′	
ECH_0944	RRG2064	Forward: 5′-GGTTTGCCCTATCAGGGTATC-3′	426bp
	RRG2065	Reverse: 5′-CACCAGACATTGACTCTTCATCT-3′	

**Table 3:** Primers used for the recombinant plasmids.

Target	Primer	Sequence ^#^	Amplicon Size	Vector
ECH-0179	RRG2097	Forward: 5′-tgacg-GGATCC-TAGTGCCATTGGAGTATATGTAAG-3′	1976 bp	pBluescript II SK(+)
	RRG2098	Reverse: 5′-tgacg-GTCGAC-TTTACTTTAATTTTAATAAAGCTGCTG-3′		
ECH_0328	RRG2099	Forward: 5′-tgacg-GTCGAC-TCTTAATCTATAAGCGGCATATGC-3′	1893 bp	pBluescript II SK(+)
	RRG2100	Reverse: 5′-tgacg-GGATCC-AATATAGGAACAACTAAACAAACTAC-3′		
ECH_0379	PRG2131	Forward: 5′-tgacg-GGATCC-GTTTTTTAGCATCCTTTGTGTTAAAAG-3′	1543 bp	pBluescript II SK(+)
	PRG2132	Reverse: 5′-tgacg-GTCGAC-ATATCGACAAGCAATTGATACAGAG-3′		
ECH_0466, to 0469	RRG2101	Forward: 5′-tgacg-GGATCC- ATGTAGAATTCACAGAGCTTTAGC-3′	2291 bp	pBluescript II SK(+)
	RRG2102	Reverse: 5′-tgacg-GTCGAC-ATTATGATTTGACACTAGACTTACAC-3′		
ECH_0474	RRG2103	Forward: 5′-tgacg-GGATCC-TGTTGTTGATATATATGTTCAGTATG-3′	1883 bp	pBluescript II SK(+)
	RRG2104	Reverse: 5′-tgacg-GTCGAC -TTACTCTGCATCTTTTGGATTACA-3′		
ECH-0944	RRG2105	Forward: 5′-tgacg-GGATCC-GTAATACAACCCCAGTTATACAGA-3′	828 bp	pBluescript II SK(+)
	RRG2106	Reverse: 5′-tgacg-GTCGAC-TAATCACTTGTCAGATTTAATGACA-3′		
*nhaA*	PRG2158	Forward: 5′-tgacg-GTCGAC-GCTCATTTCTCTCCCTGATAACA-3′	1817 bp	pBluescript II SK(+)
	PRG2159	Reverse: 5′-tgacg-CTGCAG-TGCTCTCTTCTCCTTGACCTT AC-3′		
ECH_0379	RRG1397	Forward: 5′-gag-GCTAGC-ATGATAATAAAGTTTGGGGTTGAAAATCTGATC-3′	1056 bp	pET-28a(+)
	RRG1398	Reverse: 5′-cga-CTCGAG-CTATAAATCTACACTTTCTTCAACAATATTAATAC-3′		

# Underline indicated the enzyme site.

## Data Availability

Not applicable.

## Supplementary Materials

The following are available online at https://www.mdpi.com/article/10.3390/ijms22168420/s1.
