# Supplementary material for: Functional Characterization of Multiple Ehrlichia chaffeensis Sodium (Cation)/Proton Antiporter Genes Involved in the Bacterial pH Homeostasis
Source: Int J Mol Sci. 2021 Aug 5;22(16):8420. doi: 10.3390/ijms22168420 (PMC8395091; doi:10.3390/ijms22168420)
Supplement: Supplementary file 1 [file ijms-22-08420-s001.zip › ijms-1304365-supplementary.pdf]

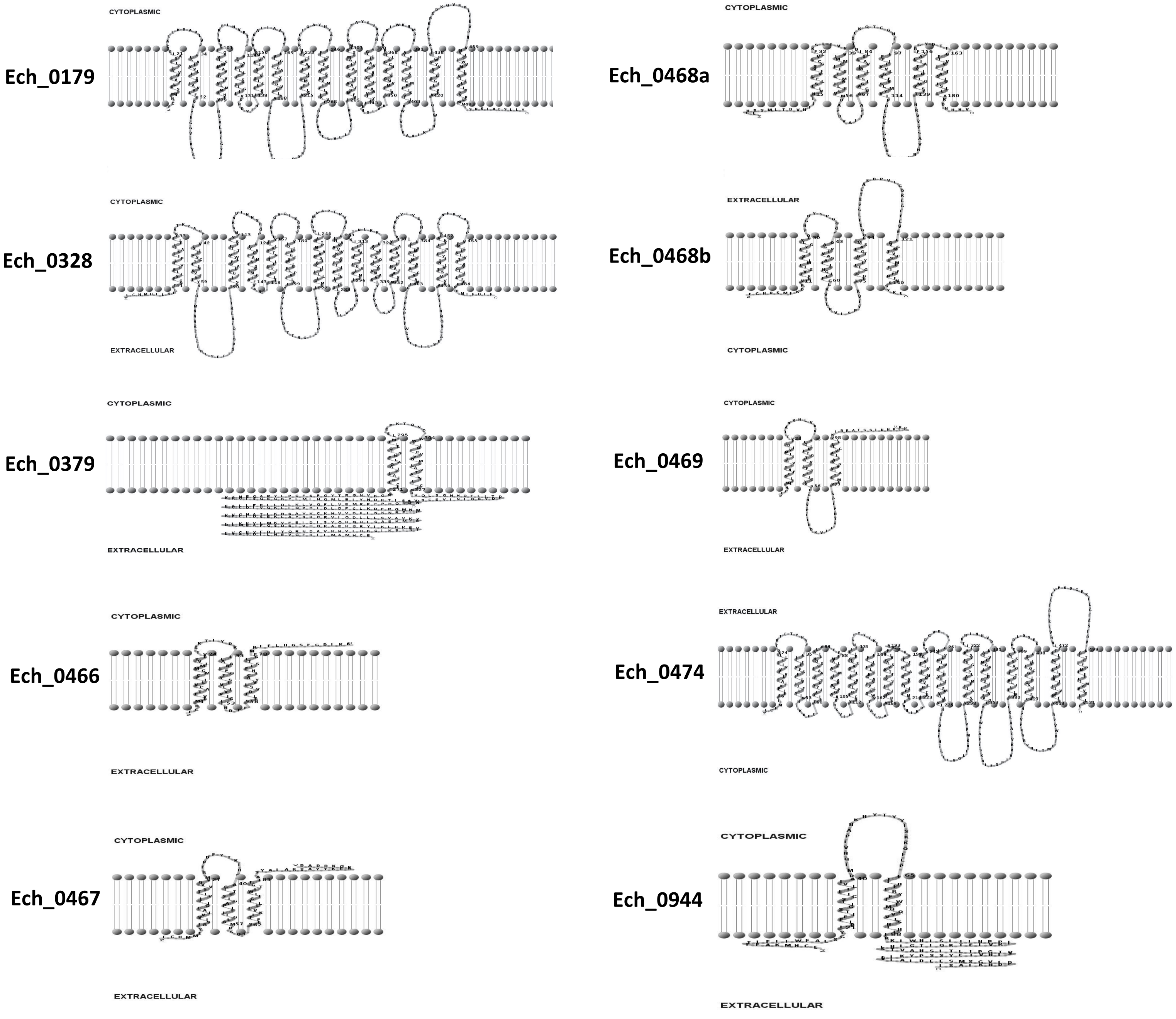

Figure S1. Prediction of transmembrane domains in *E. chaffeensis* antiporters

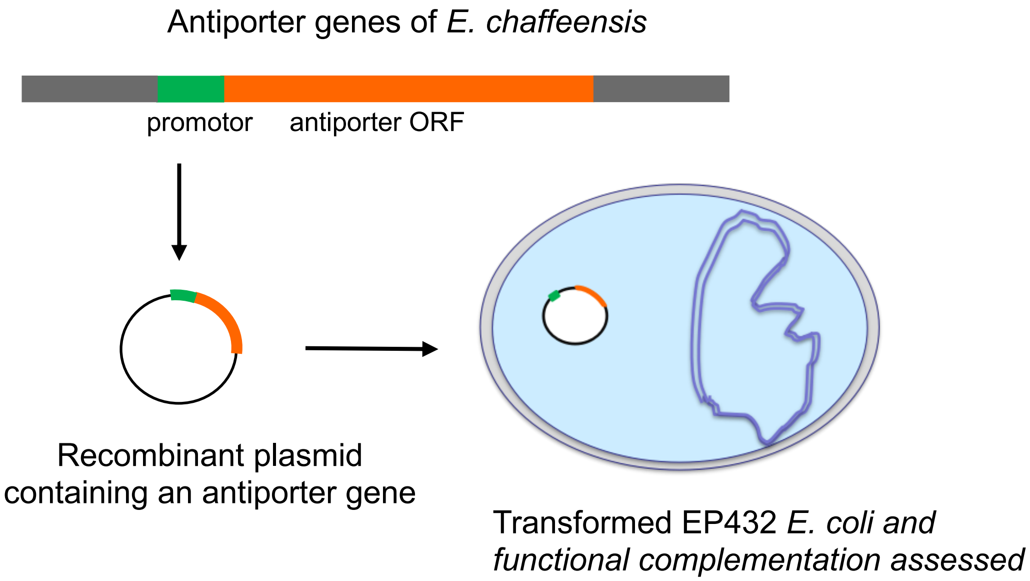

Figure S2: A cartoon depicting the *E. coli* function complementation system used in defining *E. chaffeensis* antiporters
